# Mutational hotspots and conserved domains of SARS-CoV-2 genome in African population

**DOI:** 10.1186/s43088-021-00102-1

**Published:** 2021-02-04

**Authors:** Olabode E. Omotoso, Ayoade D. Babalola, Amira Matareek

**Affiliations:** 1 Cancer Research and Molecular Biology Laboratories, Department of Biochemistry, University of Ibadan, Ibadan, Nigeria; 2 Faculty of Pharmacy, Mansoura University, Mansoura, Egypt

**Keywords:** SARS-CoV-2, Mutations, Conserved regions, Vaccine, Drug target, Virulence, Viral invasion, Coronavirus, Genome

## Abstract

**Background:**

Since outbreak in December 2019, the highly infectious and pathogenic severe acute respiratory syndrome coronavirus 2 (SARS-CoV-2) has caused over a million deaths globally. With increasing burden, the novel coronavirus has posed a dire threat to public health, social interaction, and global economy. Mutations in the SARS-CoV-2 genome are moderately evolving which might have contributed to its genome variability, transmission, replication efficiency, and virulence in different regions of the world.

**Results:**

The present study elucidated the mutational landscape in the SARS-CoV-2 genome among the African populace, which may have contributed to the virulence, spread, and pathogenicity observed in the region. A total of 3045 SARS-CoV-2 complete protein sequences with the reference viral sequence (EPI_ISL_402124) were mined and analyzed. SARS-CoV-2 ORF1ab, spike, ORF3, ORF8, and nucleocapsid proteins were observed as mutational hotspots in the African population and may be of keen interest in understanding the viral host relationship, while there is conservation in the ORF6, ORF7a, ORF7b, ORF10, envelope, and membrane proteins.

**Conclusions:**

The accumulation of moderate mutations (though slowly), in the SARS-CoV-2 genome as seen in this present study, could be a promising strategy to develop antiviral drugs or vaccines. These antiviral interventions should target viral conserved domains and host cellular proteins and/or receptors involved in viral invasion and replication to avoid a new viral wave due to drug resistance and vaccine evasion.

**Supplementary Information:**

The online version contains supplementary material available at 10.1186/s43088-021-00102-1.

## Background

The novel coronavirus infection was first reported in December 2019 in Wuhan, China. The viral infection caused by severe acute respiratory syndrome coronavirus 2 (SARS-CoV-2) was initially referred to as the Wuhan seafood market pneumonia virus before it was officially named by World Health Organization (WHO) on February 12, 2020, as coronavirus disease 2019 (COVID-19). As of January 2, 2021, 05:07 GMT, COVID-19 has spread in over 216 countries with 84,382,536 confirmed cases and 1,835,389 deaths, out of which 2,800,709 confirmed cases and 66,224 deaths were reported in Africa [1–3]. COVID-19 burden differs across regions and countries [3]; primarily due to incidence of index cases, countries’ demographic structure, life expectancy, level of adherence to public health guidelines, and the measures put in place to curtail sporadic community transmission [4, 5].

**Fig. 1:**
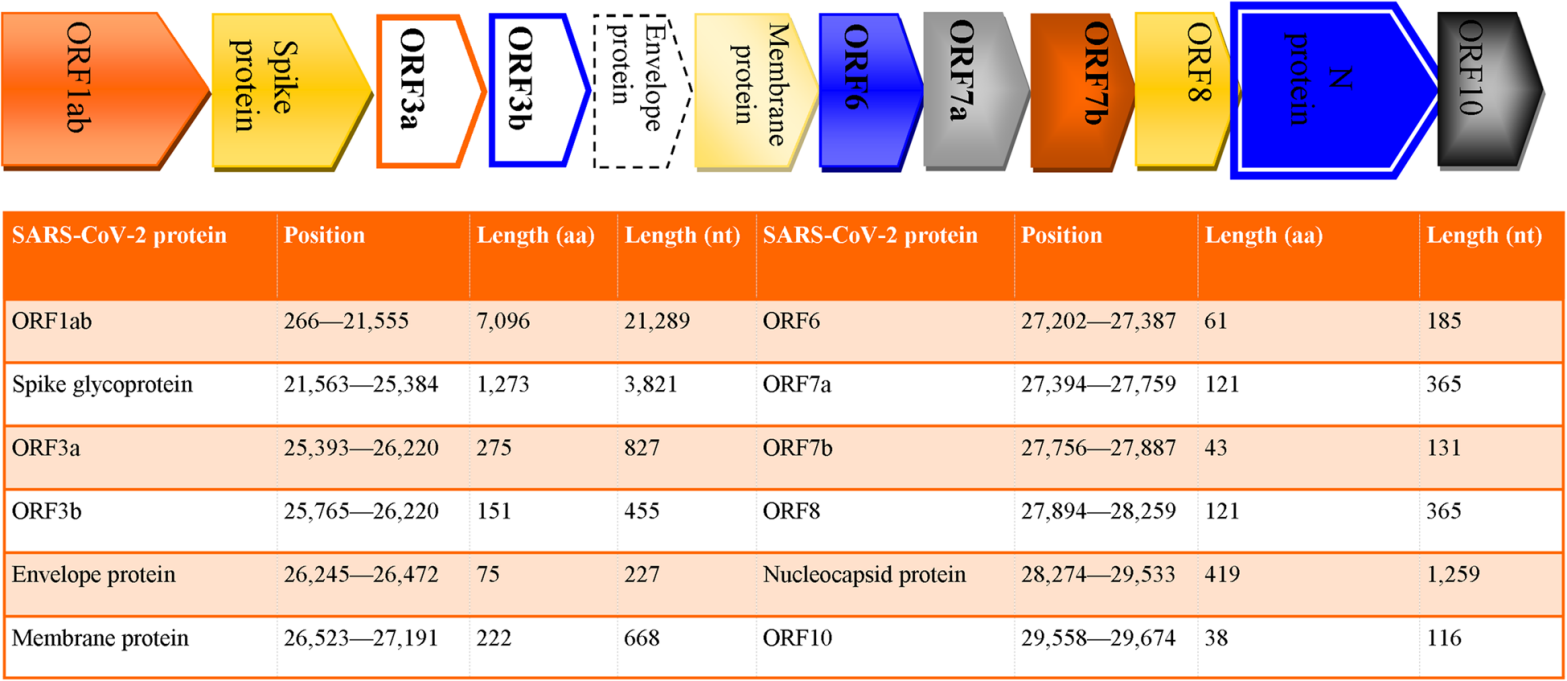
Schematic representation of SARS-CoV-2 genome with corresponding encoded proteins, their position, and length

Coronaviruses have the largest genome (about 26 to 32 kb) among all RNA viruses. It encodes *ORF1ab*, *ORF3a*, *ORF6*, *ORF7a*/b, *ORF8*, *spike* (S), *envelope* (E), *membrane* (M), and *nucleocapsid* (N) gene (Fig. 1) [6, 7]. The clade S (ORF8 variant—L84S), clade V (ORF3a variant—G251V), and clade G (spike protein variant—D614G) are the most predominant clades of the novel coronavirus [8] as characterized by the Global Initiative on Sharing All Influenza Data (GISAID). Although an earlier study [9] confirmed a moderate SARS-CoV-2 mutation, the question still remains if viral transmission and burden observed in different regions could be attributed to geographic distributions of viral gene variants.

Comprehensive genomic analysis of epidemiologic viral sequences can be a promising approach to understanding SARS-CoV-2 transmission and pathogenesis. Thus, genomic variability studies of SARS-CoV-2 can help in gaining insight into its genomic diversity in order to adopt measures in curtailing the menace of COVID-19. The efforts of researchers, WHO, National Center for Biotechnology Information (NCBI), and GISAID in making scientific reports and SARS-CoV-2 genome sequences publicly available has aided rapid understanding of viral transmission and the host-viral mechanism of action [10, 11]. Vaccines over the years have been found helpful in saving millions of lives from infectious diseases globally [12].

Previous studies [4, 10, 11, 13, 14] on SARS-CoV-2 mutational analysis have been helpful to scientists, health organizations, and WHO among many others who are working vehemently toward finding effective and potent vaccine(s) to protect against COVID-19. Based on these efforts, there are presently over 50 COVID-19 vaccines in trial [12]. Though earlier reports as aforementioned have examined the mutational landscape of SARS-CoV-2 on a global scale, our study is the first to stratify viral samples from Africa at this magnitude (in terms of viral sample size and number of African countries sampled). Due to the persistent accumulation of SARS-CoV-2 mutations [13] and to ensure Africa’s mutational landscape is adequately sampled, this present study contributes to the ongoing effort against COVID-19 by stratifying SARS-CoV-2 genomic conservation and variations in the African population. These findings will assist in understanding the pattern of viral transmissibility and virulence in Africa and in contributing to the recent development of vaccine and drug design.

## Methods

### Data acquisition

We used a slightly modified methodology [15]. A total of 5229 SARS-CoV-2 whole-genome protein sequences of African origin were assessed on the GISAID database (https://epicov.org/epi3) on January 2, 2021. A total of 3045 SARS-CoV-2 complete protein sequences from 27 African countries (who sequenced and submitted viral samples to the GISAID) were mined, after filtering as all lineage and clade, complete, *Homo sapiens* as host, high coverage only, collection and submission date of the viral sample (December 2019-January 2, 2021), and originating from Africa, while low coverage sequences (> 5% NNNs) which might be the product of sequencing errors were excluded. Comprehensive geographic information, the laboratory where the viral samples were sequenced, and accession numbers of SARS-CoV-2 genomic sequences used in the present study are provided as Supplementary file S1.

### Sequence and mutational analysis

In the present study, the mined 3045 SARS-CoV-2 datasets were analyzed with respect to the reference sequence WIV04 (EPI_ISL_402124) using the EpiCoV web interface (https://epicov.org/epi3). Viral sequences with incomplete genomic sequence, low coverage, undetermined residues (labeled as X), and genomic sites with few or single mutations were excluded. While recurrent mutations were focused on as they are likely candidates for SARS-CoV-2 transmissibility, adaptability to the human host, and possible target for drug and vaccine development. The viral sequences were classified into clades (G, GH, GR, GV, L, O, S, and V) based on the mutational distribution. Mutation frequencies were presented numerically and as percentages using basic arithmetic operations.

## Results

Our analysis of 3045 SARS-CoV-2 sequences from 27 African countries studied highlights high rate recurrent mutations observed at the following genomic sites in the SARS-CoV-2 ORF1ab polyprotein site: nsp2 region; T265I (197 viral sequences), 3 chymotrypsin-like proteinase region (3CL^pro^); G3278S (191 viral sequences), nsp6; L3606F (152 viral sequences), and RNA dependent RNA polymerase (RdRp) region; P4715L. Mutations at RdRp P4715L occurred in 2787 viral sequences (91.5%) flagging the RdRp region as a SARS-CoV-2 mutational hotspot. The nsp2 I739V and P765S variants were simultaneously observed in 12 viral sequences, predominantly from Nigeria (66.67%). More gene variants were observed in the ORF1ab non-structural proteins; nsp2, 3CL^pro^, and RdRp.

Recurrent mutations were observed in the SARS-CoV-2 spike glycoprotein; A222V and S477N variants in 17 viral sequences, respectively. One hundred three viral sequences (all originating from South Africa) carry the N501Y mutation. The spike D614G mutation was observed in 2881 viral sequences (94.6%), flagging this position as a SARS-CoV-2 mutational hotspot in Africa. Highly recurrent mutations were also observed in the SARS-CoV-2 ORF3 protein: Q57H and G251V mutations in 383 and 22 viral sequences, respectively. The prevalence of the ORF3 Q57H missense mutation in South African samples is relatively low (6.4%) with a relatively high incidence in Egypt (62.7%).

The ORF8 L84S variant was observed in 169 viral sequences with very few/no observance in viral sequences from Southern and Central Africa. Recurrent mutations in the viral genome are presented in Table 1 and overall mutational distribution across the SARS-CoV-2 genome in Africa is presented as Supplementary file S2. In the N phosphoprotein, missense mutations were observed in P13L (9 viral sequences), S194L (188 viral sequences), R203K (1539 viral sequences), and G204R (1516 viral sequences). Of the 188 N protein S194L mutations observed, 177 (94.1%) were observed from South African viral samples. More so, the recurrent R203K and G204R mutations were observed in most viral sequences from South Africa (80.6%). There was a relative conservation of the SARS-CoV-2 ORF6, ORF7, ORF10, envelope protein, and the membrane glycoprotein. The mined viral sequences were characterized according to their genetic diversities in clades: GR—1534 (50.4%), G—895 (29.4%), GH—372 (12.2%), S—166 (5.5%), O—45 (1.5%), V—16 (0.5%), GV—14 (0.5%), and L—3 (0.1%).

**Table 1:** Recurrent mutation distribution in the SARS-CoV-2 genome in African population

		S protein		N protein			ORF3	ORF8	nsp2	nsp5	nsp6	nsp12
Country	Viral samples	N501Y	D614G	S194L	R203K	G204R	Q57H	L84S	T265I	G3278S	L3606F	P4715L
Global	230,845	607	214,571	13,479	77,609	76,904	49,109	6142	31,802	4923	15,027	214,154
Africa	3045	103	2881	188	1539	1516	383	169	197	191	152	2787
Algeria	3		3				3		3			3
Benin	12		8				5	4	5			7
Botswana	1		1				1		1			1
Burkina Faso	50		38		13	13	3	32				18
Congo	8		8				7					8
Côte D’Ivoire	43		41				10	28	3		5	15
DR Congo	186		178		16	16	14	4		7	35	178
Egypt	150		143	3	24	3	94	6	2	12		94
Equatorial Guinea	45		45	1							2	45
Gabon	4		2				1	2	1			2
Gambia	235		234		83	83	16		12		3	234
Ghana	15		7		3	3	2	8				7
Kenya	25		19		2	2	1	3	1		3	19
Madagascar	6		4		2	2	2				2	4
Mali	22		7				6	13	3		2	8
Morocco	59		59		22	22	14		10		1	59
Mozambique	2		2		1	1	1		1			2
Nigeria	140		120		66	65	41	11	9	2	13	121
Reunion	4		4				1		1			4
Rwanda	5		5	1			1					5
Senegal	83		77		14	14	16	4	12		5	77
Sierra Leone	10		5		2	2	2	4	2		1	5
South Africa	1735	103	1730	177	1241	1240	111	1	111	160	60	1730
Tunisia	43		39	1	8	8	20	2	9	2	5	39
Uganda	121		64	1	14	14	5	47	5	8	13	64
Zambia	1		1		1	1						1
Zimbabwe	37		37	4	27	27	6		6		2	37
Total	3045	103	2881	188	1539	1516	383	169	197	191	152	2787

3045 complete SARS-CoV-2 sequences from 27 African countries were mined and analyzed (all recurrent mutations observed are presented as Supplementary file S2)

*Highly recurrent mutations were observed in ORF1ab polyprotein (nsp 5, 6, and 12), S protein (S1 domain), ORF3, and N protein, flagging these regions as SARS-CoV-2 mutational hotspots

## Discussion

The genome, being the molecular architecture of life encodes its phenotypic and genotypic expression. Evolving SARS-CoV-2 gene variants play a significant role in its replication, spread, and pathogenicity with respect to its human host [14]. The present study assessed SARS-CoV-2 genomic variability in the African population to understand the epidemiology, viral-host relationship, and resultant effect of such mutations. We also identified conserved domains as loopholes in the SARS-CoV-2 genome as potential targets for vaccine development and/or drug design. We identified ORF1ab RdRp and RNA primase, S, ORF3, ORF8, and N proteins as SARS-CoV-2 mutational hotspots with a conserved E, M, ORF6, ORF7a/b, and ORF10 proteins.

Generally, the clade GR—1534 (50.4%), and G—895 (29.4%) characterized by the spike D614G, nucleocapsid R203K, and G204R variants were the most prevalent in our study. Clades G and GR from previous reports [10, 16] have been mostly observed in Europe. Our study corroborates the WHO report [17] whereby most index case of COVID-19 in Africa was from Europe and North America instead of Asia, where it originated. Earlier findings [4, 10] have also indicated the prevalence of the G and GR clade in viral sequences originating from Africa.

The leader protein, ORF1ab, cleaved into nonstructural proteins (nsp1-nsp16) is essential for genome replication. RdRp is responsible for viral RNA replication, thus, due to this important role, it is expected that RdRp is well conserved. Interestingly, the present study corroborates earlier findings [11, 14] with reported recurrent missense mutations in the RdRp region resulting in protein sequence alteration. In particular, the RdRp P4715L mutation (observed in 2787 viral sequences) located close to a hydrophobic cleft has been identified as a potential antiviral drug target [11]. As of January 2, 2021, the P4715L mutation has been observed in 214,154 (92.8%) viral sequences and the T265I mutations in 31,802 (13.8%) viral sequences globally (https://epicov.org/epi3). Due to its high binding affinity (Kd 21.83 nM) to RdRp, Atazanavir has been identified as a potential COVID-19 therapeutic candidate [18]. Interestingly, the RdRp P4715L and S protein D614G mutation co-evolved in the same viral sequences (96.5%); connoting a synergistic effect of these two (2) hotspot mutations [10, 16].

The 3CL^pro^ enzyme plays a vital role in the SARS-CoV-2 life cycle, replication, and processing of the carboxyl-terminus of nsp4 through nsp16 [19]. The 3CL^pro^ was a candidate antiviral drug target during the outbreak of Middle East respiratory syndrome coronavirus (MERS-CoV) and SARS-CoV. Molecular docking application has identified aliskiren, dipyridamole, mopidamol, and rosuvastatin as potential antiviral candidates due to their relatively high binding energy to the ORF1ab 3CL^pro^ domain [18]. The significance and the relative conservativeness of the SARS-CoV-2 3CL^pro^ make it a suitable antiviral target as reported in previous studies [19, 20].

The S glycoprotein mediates host cell-surface receptor binding via its S1 domain and induces host-membrane fusion through the S2 domain. This suggests its important role in viral-host tropism, transmission, and invasion [21]. Several COVID-19 vaccine candidates approved or in clinical trial are inactivated or live-attenuated viruses, or those that target the SARS-CoV-2 S protein [8, 12]. The novel spike N501Y mutation detected in 607 viral sequences globally, found in both the 501Y.V2 and SARS-CoV-2 VOC 202012/01 was only observed in 103 viral sequences originating from South Africa and not in any other African countries studied. To gain cellular entry, the S glycoprotein [21] binds to the human angiotensin-converting enzyme (ACE) 2 receptor, facilitating human transmission. Hence, variations in this region may have a significant effect on viral fitness due to decreased binding affinity for the host ACE2 protein. In a bid to evade host-immune response, the S glycoprotein being a surface protein is constantly under selective pressure; this might explain the observed recurrent mutations in this domain in order to promote its adaptation to the host genome. Mutations in the SARS-CoV spike S1 domain give it a selective advantage in binding much more tightly to human ACE2 compared to civet SARS-CoV S1 [21]. The present study observed a highly recurrent mutation (D614G) in the spike S1 domain with a relatively conserved S2 domain, which may infer viral-host membrane fusion as the central function of SARS-CoV-2 S glycoprotein. An earlier report established that coronaviruses can elicit receptor-independent entry into host cells [21]. Therefore, preference should be given to understanding the mechanism of S2 domain mediating host-cell membrane fusion as potential cellular targets for antiviral interventions [18].

The N phosphoprotein composed of the carboxyl- and N-terminal domain forms the ribonucleoprotein complex with the viral RNA; which enhances viral genome transcription, facilitates helical nucleocapsid formation, and membrane protein interaction during virion assemblage [19, 22]. Gene variants in the N domain alter its binding to miRNAs, which might contribute to the pathogenesis and progression of infection in COVID-19 patients [14]. However, despite its ability to elicit an immune response, no N-targeted COVID-19 vaccine has been reported [8]. The N protein S194L variant was predominant in viral samples originating from South Africa (94.1%). Except for viral sequences from Egypt, Northern Africa, N protein R203K, and G204R mutations simultaneously occurred in the same viral sequences, this explains a synergistic function of these mutations. The most frequently mutated S protein D614G co-evolves with other recurrent mutations (RdRp P4715L, N protein R203K, and G204R mutations) [19]. These co-mutations are present in critical protein regions which facilitate viral ACE-2 host-entry, RNA replication, and virion assemblage. These co-mutations might confer higher viral-host transmissibility [19].

The M protein which consists of three transmembrane domains determines the shape of the viral envelope, while the E protein facilitates viral assemblage and budding [22]. The interaction of S glycoprotein with M protein is necessary to retain S protein in the Endoplasmic Reticulum-Golgi intermediate compartment/Golgi complex after membrane fusion and its integration into new virions [22]. The M protein also binds to N phosphoprotein to stabilize the nucleocapsid and aid viral assembly. During viral replication, E protein is upregulated in the infected cell facilitating viral assembly. The role of E protein in viral maturation has been expressed in E protein knock-out recombinant coronaviruses, with resultant crippled viral maturation and reduced viral titers [22]. Despite the conservation of the SARS-CoV-2 E and M proteins observed in our study, due to their small molecular size and poor immunogenic activity for humoral responses, they are yet to be explored alone as suitable COVID-19 vaccine target [8].

There is no substantial report to attribute the involvement of ORF10 in SARS-CoV-2 transmission and pathogenesis. The viral ORF3 and ORF10 proteins can synergistically attack heme on the host’s hemoglobin 1-β chain, thereby disintegrating iron to form porphyrin. This will result in reduced levels of hemoglobin carrying oxygen and carbon dioxide, interfering with the heme pathway, extreme poisoning, and inflammation of the hepatocytes [18]. Studies on chloroquine (CQ) and hydroxychloroquine (HCQ) antiviral mechanism of action depicts their inhibitory activities against viral S protein and ORF8 binding to porphyrin. They also inhibit the viral ORF1ab, ORF3, and ORF10 proteins attacking heme to form porphyrin, thus easing respiratory distress symptoms [18]. The use of CQ and HCQ as a potent drug against coronavirus has generated controversies due to their adverse effects on patients. The US Food and Drug Administration on Jun 16, 2020, retracted the use of CQ and HCQ as potent therapeutic candidates for coronavirus treatment due to their lack of efficacy and safety concerns (http://www.chinadaily.com.cn/). Hence, the quest for a clinically approved and efficient therapeutic agent is still on and our study has been able to suggest potential targets for drugs or vaccine development.

Currently, the Pfizer-BioNTech, Moderna, and AstraZeneca’s COVID-19 vaccines have been authorized and recommended for use, while the Janssen and Novavax, among many other COVID-19 vaccines Phase 3 clinical trials are being planned or currently in progress [23]. However, with the advent of the SARS-CoV-2 B.1.1.7 variant that has spread across 33 countries, there has been a concern of vaccine efficacy and evasion. This calls for continuous surveillance of the SARS-CoV-2 genome through mutational studies. Due to our keen interest in mutations that affect protein sequence, synonymous mutations which do not alter amino acid residue were not accounted for in the present study. More so, this genomic dataset includes very few viral sequences (< 50) from most of the African countries (67%) sampled (all countries sampled are presented in Table 1 in alphabetical order), while some African countries do not have any viral sequences originating from them available in recognized public repositories. Therefore, some African countries’ gene variants might likely remain unsampled. Hence, we encourage support for biomedical researchers and research institutes in developing countries in order to generate extensive genomic resources to understand viral transmissibility, evolution, and variation in the African region.

## Conclusion

SARS-CoV-2 genomic instability shows that antiviral drugs targeting the viral proteins might not be as potent as drugs targeting the host’s cellular proteins and/or receptors. Hence, developing antiviral interventions with respect to the viral conserved domains and host proteins involved in viral invasion and replication might be a promising strategy. Africa is blessed with traditional plants that have been used over the years in managing and treating a wide spectrum of diseases; hence, the combination of traditional African medicine and other candidate antiviral drugs might have better therapeutic prospects. No doubt, the traditional plants and candidate medicines will require extensive clinical trials to ascertain the safety concerns, mechanism of action, adverse effects, and efficacy.

## Supplementary Information

**Additional file 1.** Supplementary file S1.

**Additional file 2.** Supplementary file S2.

## Data Availability

All data used and generated in the course of the study are submitted as supplementary files.

## Abbreviations

3CLpro: 3 chymotrypsin-like proteinase
ACE: Angiotensin-converting enzyme
COVID-19: Coronavirus disease 2019
CQ: Chloroquine
E: Envelope protein
GISAID: Global Initiative on Sharing All Influenza Data
HCQ: Hydroxychloroquine
M: Membrane protein
MERS-CoV: Middle East respiratory syndrome coronavirus
N: Nucleocapsid
nsp: Nonstructural proteins
RdRp: RNA dependent RNA polymerase
S: Spike glycoprotein
SARS-CoV: Severe acute respiratory syndrome coronavirus
UTRs: Untranslated regions
VOC: Variant of concern
WHO: World Health Organization
